# Clonal yet Different: Understanding the Causes of Genomic Heterogeneity in Microbial Species and Impacts on Public Health

**DOI:** 10.1128/mSystems.00097-19

**Published:** 2019-05-07

**Authors:** Cheryl P. Andam

**Affiliations:** a Department of Molecular, Cellular and Biomedical Sciences, University of New Hampshire, Durham, New Hampshire, USA

**Keywords:** bacterial pathogens, microbial evolution, microbial population genomics

## Abstract

Why are members of a microbial species not the same? They may be clonal, but microbial populations are often composed of multiple cocirculating lineages distinguished by large phenotypic and genetic differences. Species and the mechanisms of speciation have been notoriously challenging to study in microbes owing to pervasive horizontal gene flow, widespread geographical distribution, and cryptic ecological niches that structure microbial populations. Understanding the origins of genomic variation in microbial species and populations is fundamental to questions critical to society and public health, such as “Are emerging diseases new species or variants of existing ones?,” “What makes a resistant strain successful?,” and “How will a pathogen respond to selective pressures?” To explore these questions, I use whole-genome sequencing of closely related strains to understand the evolutionary, ecological, and epidemiological dynamics of bacterial pathogens to inform effective, more precisely targeted public health interventions.

## Abstract (precis)

Why are members of a microbial species not the same? They may be clonal, but microbial populations are often composed of multiple cocirculating lineages distinguished by large phenotypic and genetic differences. Species and the mechanisms of speciation have been notoriously challenging to study in microbes owing to pervasive horizontal gene flow, widespread geographical distribution, and cryptic ecological niches that structure microbial populations.

## PERSPECTIVE

Whole-genome sequencing of microbial isolates has proven invaluable in infectious disease surveillance and response, particularly in elucidating a pathogen’s circulation dynamics, transmission linkages, spread of antibiotic resistance, localized outbreaks, or geographical patterns of dissemination. Genomic data, however, have revealed that although they may be clonal, microbial species and populations are often composed of multiple cocirculating lineages distinguished by phenotypic and genetic differences, the latter originating from both allelic variation and gene content variation (1). The existence of high levels of genomic heterogeneity within the same species is puzzling and represents a fundamental gap in our knowledge of microbial ecology, species formation, and evolutionary relationships. This is because, until recently, it has been difficult to explore the mechanisms of speciation and the processes that give rise to individual-level patterns of variation at the microbial scale. Some of the reasons cited to explain the challenge of studying microbial species include their extraordinary ability to transfer genetic material across species boundaries, widespread geographical distribution, and obscure ecological niches that can structure microbial populations.

Filling in this gap in our knowledge is particularly important in our understanding of infectious diseases, specifically, how genetic diversity arises and the factors that favor the success of some lineages and not others. Population genomics, which allows us to distinguish fine-scale variations among hundreds and even thousands of very closely related strains, is a powerful tool to explore the causes, mechanisms, and consequences of genomic heterogeneity within a species (2). Within-species variation can have major consequences to public health. In my recent work on Streptococcus pneumoniae, I used population genomic methods to identify the predictors of successful resistant strains. I discovered that the postvaccine diversity in the penicillin-resistant nonvaccine serotypes circulating in the United States between 2009 and 2013 was driven mainly by the persistence of preexisting strains and serotype switching rather than through *de novo* adaptation (3). I also uncovered variation in the proportion of penicillin resistance across different regions in the country, highlighting the potential for local selection to favor certain serotypes and resistant strains to increase in frequency as the population returns to equilibrium (4). Similarly, in Asia and Africa, we discovered that pneumococcal sequence type 217 has been in circulation globally for a long time and is undergoing diversification and adaptation in different geographical regions in response to local selective pressures (5).

My long-term research goals are to elucidate how intrinsic genomic factors and changes in the environment together drive the success of bacterial pathogens and to use this knowledge to test predictions about transmission, spread, and outbreaks of infectious diseases. From S. pneumoniae, I have since broadened my research to include foodborne and zoonotic bacteria, such as Staphylococcus aureus and Salmonella enterica, that cause life-threatening diseases in humans. Under the lens of the One Health concept, which recognizes that the health of people is connected to the health of animals and the environment (6), I also investigate host adaptation and antibiotic resistance in bacterial pathogens in pets, livestock, and wildlife. Once we understand the factors favoring the emergence of new bacterial lineages or those with novel characteristics that enhance their resistance, virulence, or transmission, it will be possible to minimize them or identify those (human) populations at risk in advance, rather than retrospectively through surveillance. In my current research, I use population genomic data to investigate three important facets of microbial species and populations: (i) heterogeneity in recombination, (ii) selection of genomic variants, and (iii) ecology of microbial gene pools.

## HETEROGENEITY IN RECOMBINATION

An important process that generates within-species diversity is recombination, defined as the reassortment of genetic variation between different genomic backgrounds (7). The rate of recombination of a species is critical for estimates of mutation and genomic change and therefore of the capability of a species to respond to and adapt to selective pressures such as vaccination and antibiotic use. Current models of microbial recombination incorporate the null expectation that recombination is a homogeneous process across the species, whereby different lineages of the same species exhibit the same rates and characteristics of exchange (8). However, recent work suggests that some strains donate or receive DNA more often than others, while some strains tend to frequently and preferentially recombine with specific partners (9). Such a pair of strains or lineages exchanging DNA more often with each other than with others is said to be linked by a highway of gene sharing (9, 10). These highways of recombination are likely to represent specific lineages that function as hubs of gene flow, facilitating the rapid spread of genes associated with antibiotic resistance, host adaptation, and immune interactions (10). Our recent work also suggests that some strains act as hyperdonors or hyperrecipients of recombined DNA. Such interstrain differences may suggest the existence of differing roles of strains in a population in structuring the pan-genome and therefore the existence of genes that are potentially available to any member of the group via additive or replacement horizontal gene transfer (HGT) (11). The existence of heterogeneity in recombination rates among members of the same species is poorly understood, and therefore we still lack a coherent model for genome evolution and pan-genome dynamics that integrates variation in recombination frequencies and characteristics within a species.

## SELECTION OF GENETIC VARIANTS

The evolution of resistance and virulence is predicted to be much quicker when selecting genomic variants already present in the population rather than those requiring *de novo* adaptation (3). Hence, the standing allelic and gene content variation is particularly important in defining the baseline of potentially successful lineages and in understanding to what extent the baseline variation shifts as they encounter different selective pressures. Key gaps in our knowledge are those concerning the complexity of the genomic variants contributing to their occurrence in distinct environments, the extent in which specific variants differ by phylogenetic lineage (i.e., how much does genetic background matter?), and the mechanisms that underlie the diversification and persistence of different genomic elements in the population. In my laboratory, we investigate the distribution and dynamics of bacterial populations from multiple sites within a single host and between hosts. We also explore the selective pressures and factors that contribute to the emergence and long-term evolution of highly resistant bacterial lineages. I expect that the broad application of population genomics methods will ultimately lead to a profound understanding of the fundamental biological principles that describe the complex, long-term behaviors of microbial populations in response to different selective pressures (e.g., antibiotic use, vaccination, dietary and nutritional changes, lifestyle change, increasing host population density, host switching), which can be used as predictors of success of a pathogen.

## ECOLOGY OF MICROBIAL GENE POOLS

The remarkably large pan-genomes (i.e., totality of genes that are present in members of a group or taxon) of many microbial species give rise to the issue of whether adaptive or neutral processes drive their composition and distribution among strains (12). Previous studies have proposed that individual genes should be considered to have their own ecological niches where they thrive, which include their genomic neighborhood (7) and cryptic niches that may not be readily observable (13). Hence, we may consider a taxon’s gene pool to be shaped by genes being selected for in specific ecologies regardless of the organism which harbors them. Moreover, gene mobility has been shown to transcend ecological and geographical boundaries; hence, gene distribution may not necessarily follow the distribution of the host cell (14). We aim to explore the influence of ecology on the heterogeneous distribution and mobility of different genomic components and how this variation relates to the species formation, taxonomic assignment, and metabolic potential of any one microbial species. Similarly, because selection on gene content can be viewed at different levels, from gene to individual organism to group to the holobiont (11), perhaps we should begin thinking of the ecology and biogeography of specific genomic elements, beyond individual strains or lineages.

## FUTURE DIRECTIONS IN MICROBIAL POPULATION GENOMICS

**FIG 1:**
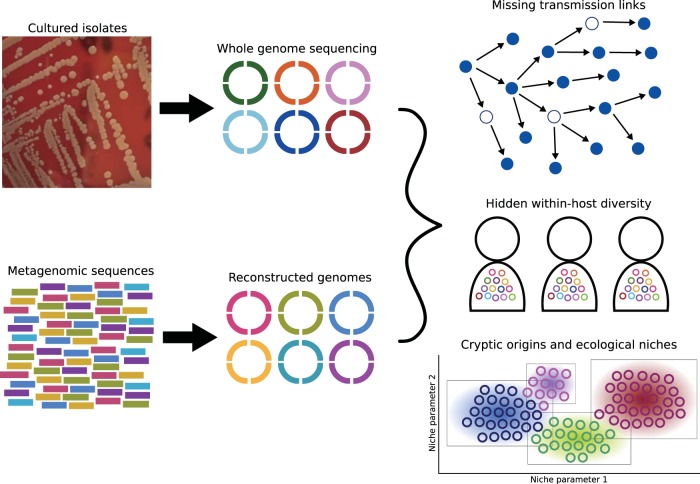
Schematic illustration of how combining whole-genome data of bacterial strains from cultivation-based and cultivation-free (metagenomics) approaches can greatly improve ecological and epidemiological investigations of infectious diseases.

Bacterial pathogens impose a heavy burden of disease on human populations worldwide, and the gravest threats are those represented by highly virulent respiratory pathogens, enteric pathogens, and HIV-associated infections (15). Microbial population genomics offers a powerful tool to help reduce the persistent global problem of antibiotic resistance and the emergence of novel virulent genotypes in these pathogens. Over the next 5 years, I expect that whole-genome sequencing of thousands of very closely related cultured strains, augmented with reconstructed genomes derived from cultivation-free approaches, will generate a more robust understanding of how microbial population dynamics and fine-scale genomic heterogeneity shape host-pathogen interactions and disease outcomes. Reconstructed genome sequences from metagenomic data will be particularly useful in defining missing links in disease transmission chains, uncovering cryptic ecological niches of emerging virulent or resistant genotypes, and precisely estimating levels of genetic diversity of a pathogen within a single host, which is often overlooked in epidemiological studies (Fig. 1). I also anticipate that microbial population genomics will be more widely utilized beyond the confines of pathogens infecting humans and will constitute a major step forward in understanding the population structure and dynamics of other microbial species in agricultural, environmental, and veterinary settings.
